# The *Xanthomonas* effector XopJ triggers a conditional hypersensitive response upon treatment of *N. benthamiana* leaves with salicylic acid

**DOI:** 10.3389/fpls.2015.00599

**Published:** 2015-08-03

**Authors:** Suayib Üstün, Verena Bartetzko, Frederik Börnke

**Affiliations:** ^1^Plant Health, Plant Metabolism Group, Leibniz-Institute of Vegetable and Ornamental Crops, GroßbeerenGermany; ^2^Division of Biochemistry, Department of Biology, Friedrich-Alexander-Universität Erlangen-Nürnberg, ErlangenGermany; ^3^Institute of Biochemistry and Biology, University of PotsdamPotsdam, Germany

**Keywords:** *Xanthomonas*, type-III effector, XopJ, avirulence, salicylic acid

## Abstract

XopJ is a Xanthomonas type III effector protein that promotes bacterial virulence on susceptible pepper plants through the inhibition of the host cell proteasome and a resultant suppression of salicylic acid (SA) – dependent defense responses. We show here that *Nicotiana benthamiana* leaves transiently expressing XopJ display hypersensitive response (HR) –like symptoms when exogenously treated with SA. This apparent avirulence function of XopJ was further dependent on effector myristoylation as well as on an intact catalytic triad, suggesting a requirement of its enzymatic activity for HR-like symptom elicitation. The ability of XopJ to cause a HR-like symptom development upon SA treatment was lost upon silencing of SGT1 and NDR1, respectively, but was independent of EDS1 silencing, suggesting that XopJ is recognized by an R protein of the CC-NBS-LRR class. Furthermore, silencing of NPR1 abolished the elicitation of HR-like symptoms in XopJ expressing leaves after SA application. Measurement of the proteasome activity indicated that proteasome inhibition by XopJ was alleviated in the presence of SA, an effect that was not observed in NPR1 silenced plants. Our results suggest that XopJ – triggered HR-like symptoms are closely related to the virulence function of the effector and that XopJ follows a two-signal model in order to elicit a response in the non-host plant *N. benthamiana*.

## Introduction

In nature, plants are continuously attacked by a broad range of potential pathogens. However, the majority of plants are resistant to most pathogen species. This form of resistance is known as non-host resistance (NHR) and can be defined as a broad-spectrum plant defense that provides immunity to all members of a plant species against all isolates of a micro-organism that is pathogenic on other plant species (Senthil-Kumar and Mysore, 2013). In order for a pathogen to be successful and cause disease it has to defeat the plant’s multilayered immune system. Before it can enter the plant tissue, the pathogen is exposed to a range of preformed physical and chemical barriers, already preventing the entry of many non-adapted pathogens at an early step. If a pathogen is able to overcome these barriers and comes into contact with the plant cell surface, it will face induced plant defenses. Surface-localized pattern recognition receptors (PRRs) can perceive conserved pathogen molecules (PAMPs, pathogen associated molecular patterns) which in case of bacteria are for instance flagellin, elongation factor Tu (EF-Tu), peptidoglycan (PGN) or lipopolysaccharides (Macho and Zipfel, 2015). This recognition results in the initiation of intra cellular down-stream signaling that leads to the production of reactive oxygen species, stimulation of mitogen-activated protein kinase (MAPK) cascades, defense gene induction, and callose deposition at the plant cell wall (Boller and Felix, 2009). These induced defense outputs are in most cases sufficiently effective to eradicate a potential pathogen from infected tissue and are collectively referred to as PAMP-triggered immunity (PTI; Jones and Dangl, 2006). As a response, several Gram-negative pathogenic bacteria use a type-III secretion system (T3SS) to inject a suite of so called type-III effector proteins (T3Es) into their eukaryotic host cell (Galan et al., 2014). These T3Es are targeted to a number of cellular compartments where they influence host cellular processes to provide a beneficial environment for the pathogen to promote pathogen multiplication and disease. In order to counter this, plants have evolved the ability to recognize specific effector proteins through resistance (R) proteins, a class of receptor proteins that typically contain nucleotide-binding domains (NB) and leucine rich repeats (LRRs), (Dodds and Rathjen, 2010). Recognition of T3Es [in that case also referred to as avirulence (Avr) proteins] by NB-LRR proteins can either be directly through physical interaction between both proteins or indirectly through an accessory protein that is part of an NB-LRR protein complex (Dodds and Rathjen, 2010). During indirect recognition, the so called guard hypothesis, it is assumed that the effector interaction is mediated by the effector target protein or a structural mimic thereof (Dangl and Jones, 2001). The activity of the T3E induces structural changes in its target protein that enables recognition by the NB-LRR protein, leading to its activation and finally results in effector-triggered immunity (ETI; Jones and Dangl, 2006). Generally, PTI and ETI give rise to similar responses, although ETI is qualitatively stronger and faster and often involves a rapid form of localized cell death called the hypersensitive response (HR) that is assumed to limit spread of biotrophic pathogens from the site of infection. The NB-LRR repertoire recognition of pathogen effector proteins is highly dynamic and depends on the genotype of a given host cultivar. Thus, ETI primarily protects against specific races of pathogens because it is only triggered when an Avr factor, i.e., a particular T3E, on the pathogen side comes together with a matching R protein on the host side. Although ETI is a major component of host/pathogen race specificity, its role in NHR is not well understood. In some cases, T3Es trigger ETI in non-host plants, suggesting a role for ETI in determining the host range of a pathogen (Staskawicz et al., 1987; Kobayashi et al., 1989; Wei et al., 2007; Wroblewski et al., 2009). The signal transduction and physiological processes leading to HR during ETI are not well understood but it appears that the defense hormone salicylic acid (SA) plays a central role in the induction of such a resistance response (Glazebrook, 2005). SA depletion in plants by transgenic expression of a bacterial SA hydroxylase encoded by *nahG* suppresses *R* gene mediated defenses elicited by a range of bacterial, oomycete, and viral pathogens (Delaney et al., 1994; Rairdan and Delaney, 2002). While in general SA is active against biotrophic pathogens some necrotrophs have acquired strategies to induce SA signaling during infection in order to promote host cell death and thus virulence. For example, the fungus *Botrytis cinerea* produces an exopolysaccharide, which acts as an elicitor of the SA pathway. In turn, the SA pathway antagonizes the jasmonic acid signaling pathway that would otherwise restrict virulence of this necrotrophic pathogen (El Oirdi et al., 2011).

The Gram-negative phytopathogenic bacterium *Xanthomonas campestris* pv. *vesicatoria* (Xcv) is the causal agent of bacterial spot disease on pepper and tomato plants (Ryan et al., 2011). During infection, it secretes a cocktail of 20–40 T3Es into the plant cell that collectively suppress defense and allow bacterial propagation (Thieme et al., 2007; White et al., 2009). Although these T3Es likely play a role in virulence in susceptible hosts, they can also have Avr function and trigger ETI in certain genotypes of pepper and tomato plants expressing cognate R proteins as well as in non-host plants from other species (Whalen et al., 1993; Bonshtien et al., 2005; Kim et al., 2010; Szczesny et al., 2012). Transient expression of T3Es by infiltration of leaves from *Nicotiana benthamiana* with *Agrobacteria* is widely used to characterize T3E virulence functions in plants (Bartetzko et al., 2009; Gurlebeck et al., 2009; Üstün et al., 2013; Stork et al., 2015). In some cases, expression of T3Es from Xcv in *N. benthamiana* has led to the induction of ETI associated with signs of an HR (Thieme et al., 2007; Schulze et al., 2012; Singer et al., 2013).

The Xcv T3E XopJ is a member of the widespread YopJ-family of effector proteins that is present among plant and animal pathogenic bacteria and whose members are highly diversified in virulence function (Lewis et al., 2011). XopJ and its close homolog HopZ4 from *Pseudomonas syringae* pv. *lachrymans* have been shown to interact with the proteasomal subunit RPT6 *in planta* to suppress proteasome activity (Üstün et al., 2013, 2014). XopJ-triggered proteasome suppression results in the inhibition of SA-related immune responses to attenuate onset of necrosis and to alter host transcription (Üstün et al., 2013). Transient expression of XopJ in leaves of *N. benthamiana* was instrumental to elucidate its function (Thieme et al., 2007; Bartetzko et al., 2009; Üstün et al., 2013). Using a XopJ-green fluorescent protein (GFP) fusion proteins a localization of the effector to the plasma membrane of the host cell mediated by myristoylation of the protein could be demonstrated (Thieme et al., 2007; Bartetzko et al., 2009). Furthermore, XopJ’s inhibitory effect on protein secretion was shown by transient co-expression of the effector together with a secretable GFP variant (Bartetzko et al., 2009). In some of these experiments XopJ was reported to elicit a cell death reaction in *N. benthamiana* 2–4 days post inoculation, suggesting recognition of the effector in this non-host plant (Thieme et al., 2007). However, this reaction was not observed in other studies (Bartetzko et al., 2009; Üstün et al., 2013). Usually, first signs of an HR become apparent within a few hours after the Avr protein is delivered to the host cell by the bacterial T3SS (Morel and Dangl, 1997). Although transient overexpression could lead to different kinetics of effector recognition in comparison to T3SS delivery, XopJ triggered cell death in *N. benthamiana* at late time points of expression could also have other reasons than weak recognition by a cognate R protein. For instance, XopJ could interfere with cellular functions requiring proteasome activity leading to a general perturbation of protein homeostasis.

In the present study, we show that XopJ elicits a rapid HR-like response in *N. benthamiana* when leaves transiently expressing the effector are sprayed with SA. Development of HR-like symptoms was closely related to XopJ’s virulence function and appears to involve indirect recognition by an R protein. A two-signal model leading to the elicitation of HR-like symptoms by XopJ in *N. benthamiana* is discussed.

## Materials and Methods

### Plant Material and Growth Conditions

Tobacco plants (*N. benthamiana*) were grown in soil in a greenhouse with daily watering, and subjected to a 16 h light: 8 h dark cycle (25°C: 21°C) at 300 μmol m^-2^ s^-1^ light and 75% relative humidity.

### Transient Expression Assays and SA Treatment

For infiltration of *N. benthamiana* leaves, *A. tumefaciens* C58C1 was infiltrated into the abaxial air space of 4- to 6-week-old plants, using a needleless 2-ml syringe. Agrobacteria were cultivated overnight at 28°C in the presence of appropriate antibiotics. The cultures were harvested by centrifugation, and the pellet was resuspended in sterile water to a final optical density at (OD_600_) of 1.0. SA treatment was performed 24 h after agro-infiltration. Infiltrated leaves were sprayed with 5 mM SA (containing 0,005% v/v Silwet-77) or water (containing 0,005% v/v Silwet-77) and phenotypes were analyZed 24 h later.

### Western Blotting

Leaf material was homogenized in sodium-dodecyl sulphate-polyacrylamide gel electrophoresis (SDS-PAGE) loading buffer (100 mM Tris-HCl, pH 6.8; 9% β-mercapto-ethanol, 40% glycerol, 0.0005% bromophenol blue, 4% SDS) and, after heating for 10 min at 95°C, subjected to gel electrophoresis. Separated proteins were transferred onto nitrocellulose membrane (Porablot, Machery und Nagel, Düren, Germany). Proteins were detected by an anti-HA-Peroxidase high affinity antibody (Roche).

### Measurement of Proteasome Activity

Proteasome activity in crude plant extracts was determined spectro-fluorometrically using the fluorogenic substrate suc-LLVY-NH-AMC (Sigma) according to Üstün et al. (2013).

### Virus-Induced Gene Silencing of *N. benthamiana*

Virus-induced gene silencing (VIGS) was performed as described previously (Üstün et al., 2012). pTRV2-SGT1, pTRV2-NPR1, and pTRV2-EDS1, pYL279-NDR1 (Liu et al., 2002) were obtained from the *Arabidopsis* Biological Resource Center (http://www.arabidopsis.org). Briefly, *Agrobacterium* strains with the pTRV1 vector and with pTRV2-GFPsil, pYL279-RPT6, (Üstün et al., 2012, 2013), pTRV2-SGT1, pTRV2-NPR1, and pTRV2-EDS1 (OD_600_ = 1.0) were mixed in a 1:1 ratio, respectively, and the mixture was infiltrated into a lower leaf of a 4-week-old *N. benthamiana* plant using a 1-mL sterile syringe without a needle. Fourteen days of post infiltration silenced plants were used for further transient expression studies.

### Ion Leakage Measurements

For electrolyte leakage experiments, triplicates of 1.76 cm^2^ infected leaf material were taken at 48 h post infiltration (hpi). Leaf disks were placed on the bottom of a 15-ml tube. Eight milliliters of deionized water was added to each tube. After 24 h of incubation in a rotary shaker at 4°C, conductivity was determined with a conductometer. To measure the maximum conductivity of the entire sample, conductivity was determined after boiling the samples for 30 min (Üstün et al., 2012).

## Results

### Treatment of *N. benthamiana* Leaves Transiently Expressing XopJ with SA Rapidly Induces Cell Death

**FIGURE 1:**
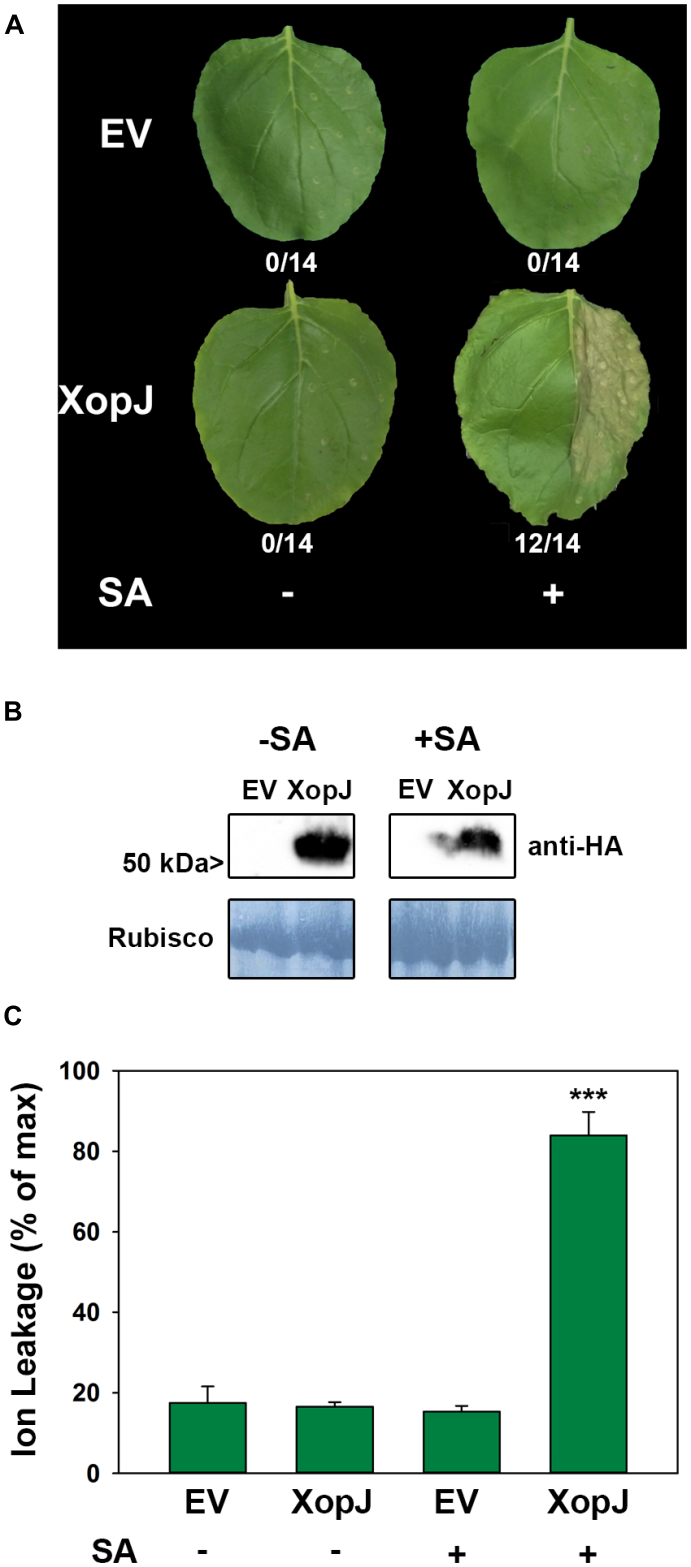
**Salicylic acid (SA) treatment of *Nicotiana benthamiana* leaves transiently expressing XopJ elicits a hypersensitive response (HR)-like response. (A)** Phenotype of *N. benthamiana* leaves (one half) infiltrated with *Agrobacterium tumefaciens* strains that mediate T-DNA-based transfer of XopJ-HA and empty vector (EV). Leaves were sprayed with 5 mM salicylic acid (or water) 24 h post infiltration (hpi) and leaves were photographed 48 hpi. The number of leaves showing HR-like symptoms from all leaves analyzed is indicated below the appropriate construct. **(B)** Protein extracts from *N. benthamiana* leaves transiently expressing XopJ-HA or EV at 48 hpi were prepared. Equal volumes representing approximately equal protein amounts of each extract were immunoblotted and proteins were detected using anti-HA antiserum. Amido black staining served as a loading control. **(C)** Ion leakage was measured in plants transiently expressing XopJ-HA and EV at 48 hpi (± SA treatment). Bars represent the average ion leakage measured for triplicates of six leaf disks each, and the error bars indicate SD. The asterisk indicates a significant difference (***P < 0.001) based on results of a Student’s *t*-test. The experiment has been repeated three times with similar results.

Previous results suggested that during a compatible interaction of Xcv with pepper XopJ exerts its virulence function by inhibiting SA-mediated defense responses (Üstün et al., 2013). However, when Xcv infected pepper leaves were treated with SA they developed necrotic lesions that were comparable to those observed on Xcv Δ*xopJ* infected leaves at the same time point without SA treatment (Üstün et al., 2013). Thus, Xcv infected tissue remains sensitive to exogenously applied SA even in the presence of XopJ. To further study the role of XopJ in interfering with SA-related processes, we sought to investigate the consequences of SA application on XopJ expressing leaves of the non-host plant *N. benthamiana*. To this end, an HA-tagged version of XopJ under control of the CaMV35S promoter (XopJ-HA) was transiently expressed in leaves of *N. benthamiana* using *Agrobacterium*-infiltration. Twenty four hours post infiltration (hpi) XopJ-HA infiltrated and empty vector (EV) infiltrated control leaves were sprayed with 5 mM SA. As shown in **Figure 1A**, XopJ-HA expressing leaves showed tissue collapse and developed necrosis 48 hpi when treated with SA. Untreated leaves showed no signs of tissue damage even in the presence of XopJ, indicating that XopJ alone is not able to trigger necrotic cell death in the time period investigated but requires exogenously applied SA. XopJ protein expression was not affected by the SA-treatment (**Figure 1B**). The observed phenotype in SA-treated XopJ expressing leaves resembles that of a HR which is usually associated with R-protein-mediated immunity triggered upon recognition of a pathogen-derived Avr protein. An HR is often preceded by an increase in electrolyte leakage in dying cells, and measurement of electrolyte leakage caused by membrane damage is a quantitative measure of HR-associated cell death (Mackey et al., 2003). Ion leakage was strongly increased in SA-treated XopJ expressing leaves as compared to the EV control (**Figure 1C**). This effect was completely dependent on SA treatment as untreated XopJ expressing tissue did not show signs of cell damage (**Figure 1C**). Previous results indicated that a myristoylation motif at the N-terminus guides XopJ to the plasma membrane and this subcellular localization as well as an intact catalytic triad is required for the effector to function (Üstün et al., 2013). Leaves expressing a XopJ(G2A)-HA protein, which is no longer myristoylated, or a catalytically inactive variant carrying a C to A substitution at position 235 [XopJ(C235A)], developed no visible signs of tissue damage when treated with SA (Supplementary Figure S1). This suggests that development of SA-dependent phenotypes requires XopJ to be fully functional. To confirm that this effect is specific to XopJ, we transiently expressed an unrelated *Xanthomonas* effector, XopS, and treated plants with 5 mM SA. No visible signs of HR-like cell death were visible on leaves either untreated or sprayed with SA (Supplementary Figure S2), indicating that the induction of tissue collapse after SA application is specific for XopJ.

### XopJ-Mediated Cell Death after SA Treatment Requires Signaling Components of R-Protein-Mediated Resistance

**FIGURE 2:**
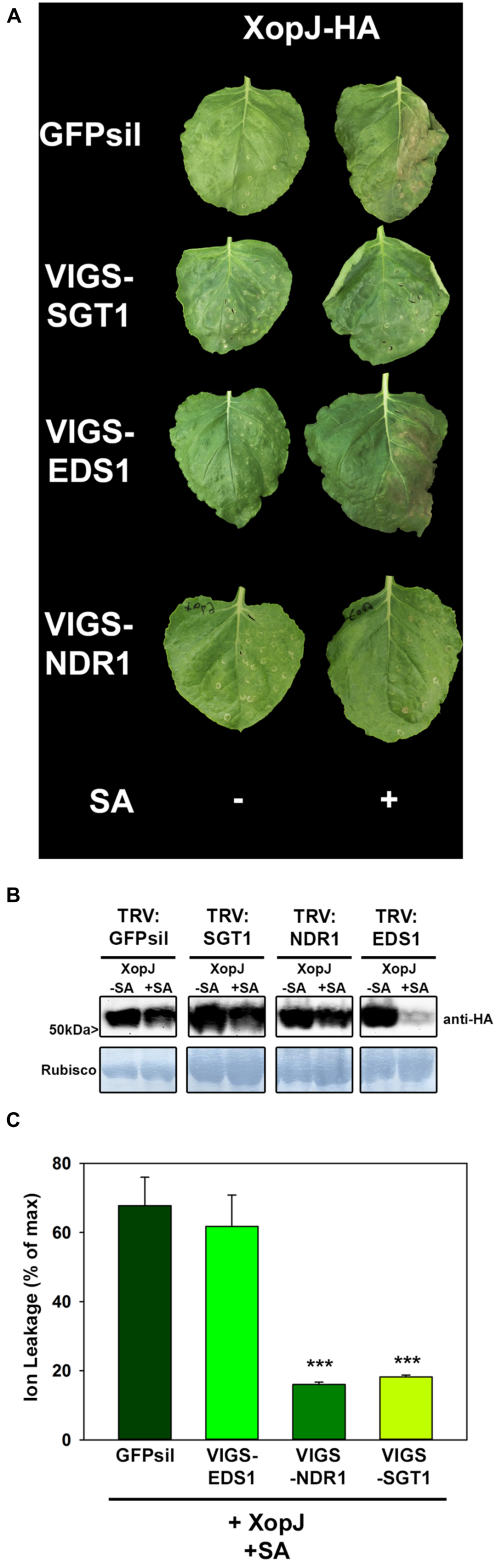
**XopJ-triggered cell-death after SA application requires components of R-protein mediated signaling. (A)** Phenotypes of *N. benthamiana* SGT1, EDS1, and NDR1 VIGS plants transiently expressing XopJ-HA and EV in comparison to the GFPsil VIGS control leaves expressing XopJ-HA and EV with or without SA treatment. Photographs were taken at 48 hpi. **(B)** Protein extracts from TRV:GFPsil, TRV:SGT1, TRV:NDR1, and TRV:EDS1 transiently expressing XopJ-HA at 48 hpi were prepared. Equal volumes representing approximately equal protein amounts of each extract were blotted onto a nitrocellulose membrane and protein was detected using anti-HA antiserum. Amido black staining served as a loading control. **(C)** Electrolyte conductivity was measured in TRV:GFPsil, TRV:EDS1, and TRV:SGT1 and TRV1:NDR1 plants transiently expressing XopJ-HA (sprayed with 5 mM SA 24 hpi) at 48 hpi. Bars represent the average ion leakage measured for triplicates of six leaf disks each, and the error bars indicate SD. Treatments were compared with TRV:GFPsil transiently expressing XopJ-HA and significant differences are indicated by asterisks (***P < 0.001). The experiment was repeated three time with similar results.

The results obtained thus far suggest that tissue collapse and necrosis upon SA treatment of XopJ expressing *N. benthamiana* leaves could involve an HR-like process and thus might be the consequence of R-protein mediated recognition of the effector triggered by SA. Defense signaling by R proteins requires further signaling components such as SGT1 (suppressor of G2 allele of skp1) which, in *N. benthamiana* was found to be required for responses mediated by a diverse range of R proteins against various pathogens (Peart et al., 2002). In order to investigate an involvement of SGT1 in XopJ-mediated cell death after SA-treatment, VIGS with Tobacco rattle virus (TRV), followed by *Agrobacterium*-infiltration and SA-treatment was used. For this purpose, young *N. benthamiana* plants (at the five-leaf stage) were infiltrated with a mixture of *Agrobacterium tumefaciens* strains of pTRV1 (CaMV 35S-driven TRV RNA1) and pTRV2-SGT1 (TRV RNA2 containing the target sequence), or pTRV-GFPsil (serving as a control for infection symptoms). Two weeks after TRV inoculation efficacy of the silencing construct was assessed by RT-PCR (Supplemental Figure S3) and silenced plants and the control were infiltrated with *A. tumefaciens* containing XopJ-HA and sprayed with SA 24 hpi. When SA-treated leaves were inspected after an additional 24 h time period, XopJ infiltrated GFPsil control plants showed clear signs of tissues collapse and necrosis while SA-treatment of XopJ expressing SGT1-silenced plants did not lead to phenotypic alterations (**Figure 2A**), although immunoblot analysis revealed XopJ protein expression levels to be similar in both plants at 48 hpi (**Figure 2B**). Measurement of electrolyte leakage showed that SA-treatment of XopJ expressing leaves caused a significant increase in cell membrane disintegration in TRV:GFPsil plants but not in TRV:SGT1 plants (**Figure 2C**), indicating that SGT1 silencing abrogates tissue damage under these conditions. These results demonstrate that XopJ requires SGT1 to elicit cell death in *N. benthamiana* upon SA-treatment. It is likely, therefore, that XopJ is recognized by a plant R-protein only after treatment of leaves with SA.

R proteins differ in their requirement for signaling components downstream of SGT1. NBS-LRR proteins with amino- terminal Toll and interleukin-1 receptor homology (TIR domain) use EDS1, whereas those with CC domains signal through NDR1 (Aarts et al., 1998). To provide first insights into the nature of a possible R protein involved in the recognition of XopJ after SA-treatment in *N. benthamiana*, VIGS directed against EDS1 and NDR1 was used (Supplementary Figure S3). Silencing of NDR1 resulted in a clear and consistent decrease in HR-like symptom development upon SA-treatment of XopJ infiltrated leaves, while plants with reduced EDS1 expression showed no apparent phenotypical differences when compared with the TRV:GFPsil control (**Figure 2A**). XopJ protein expression was confirmed by immunoblot analyses in EDS1 and NDR1 silenced leaves (**Figure 2B**). In accordance with previous findings (Üstün et al., 2012), we realized that XopJ protein levels are reproducibly lower in SA-treated *EDS1* silenced plants which might be due to an accelerated HR induction in these plants affecting the level of some proteins. Consistent with the observed phenotype, a significant decrease in ion leakage following SA-treatment of XopJ expressing leaves was evident in TRV:NDR1 plants but not in TRV:EDS1 and TRV:GFPsil plants, respectively (**Figure 2C**). This suggests that the R protein mediating the response to XopJ after SA-treatment is a member of the CC-NBS-LRR class.

### Elicitation of HR-Like Symptoms is Dependent on RPT6

**FIGURE 3:**
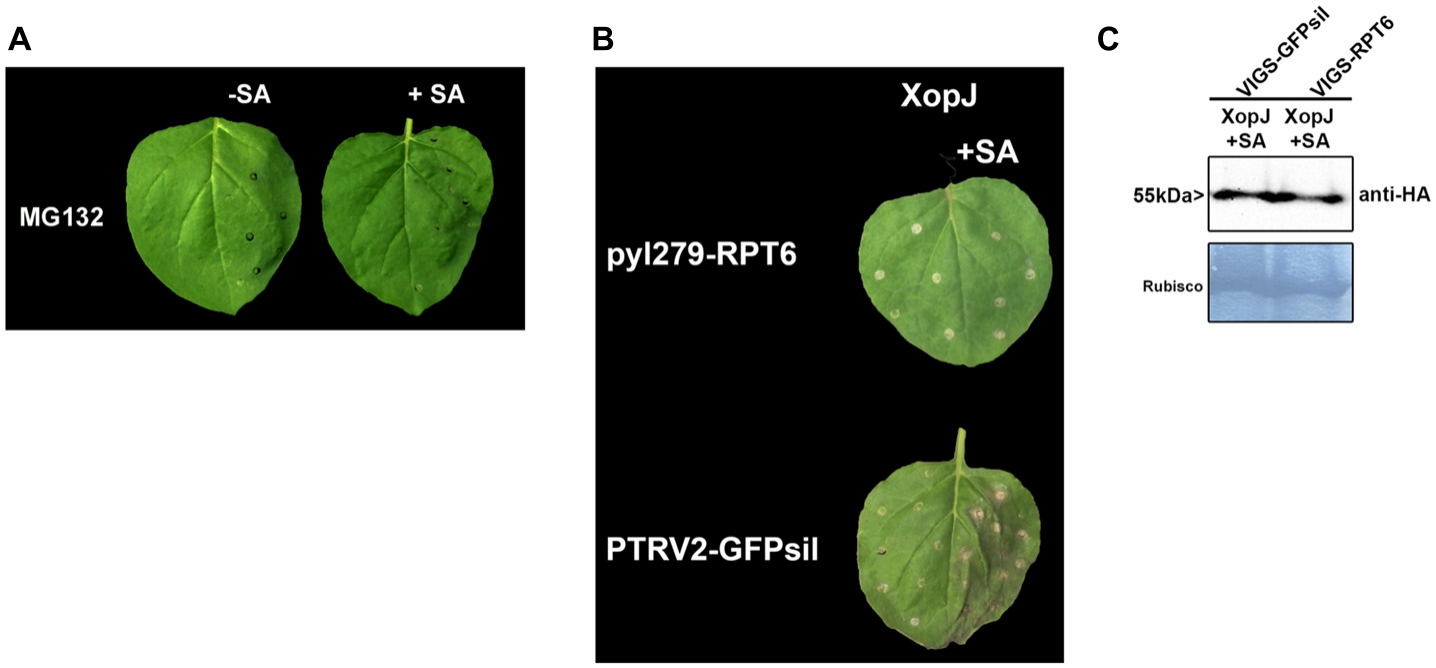
**RPT6 is required for XopJ-induced HR-like symptoms. (A)** Phenotypes of *N. benthamiana* leaves infiltrated with 100 μM MG132 with or without SA. **(B)** XopJ was transiently expressed in RPT6 silenced leaves or control plants and treated with 5 mM SA 24 hpi. Plant reactions were photographed 48 h after transient expression of XopJ. **(C)** Western blot analysis of XopJ-HA protein levels in TRV:RPT6 and TRV:GFPsil control plants using an anti-HA antibody. Amido black staining served as a loading control. The experiment was repeated three times with similar results.

XopJ acts as a protease to degrade the proteasomal subunit RPT6 in host cells (Üstün and Börnke, 2015). This results in an inhibition of proteasomal activity which finally attenuates SA-dependent defense responses (Üstün et al., 2013). In order to investigate whether inhibition of proteasome activity *per se* is sufficient to elicit HR-like symptoms upon SA treatment or whether this effect requires the action of XopJ on RPT6, *N. benthamiana* leaves were treated with the potent proteasome inhibitor MG132 6 h before SA treatment. In contrast to XopJ expressing tissue, leaves pretreated with MG132 before SA application did not develop any visible signs of HR indicating that a general inhibition of the proteasome is not sufficient to elicit this response in *N. benthamiana* (**Figure 3A**). To assess the requirement of the XopJ virulence target RPT6 for elicitation of SA-dependent HR-like symptoms, *N. benthamiana* leaves transiently expressing XopJ and silenced for *RPT6* expression using VIGS (Supplementary Figure S3) were treated with SA. As shown in **Figure 3B**, the pTRV2-GFPsil control expressing XopJ showed typical signs of cell death while RPT6 silenced plants did not develop visible symptoms. Similar levels of XopJ protein expression were observed in both types of VIGS plants (**Figure 3C**). These finding lend support to the notion that elicitation of SA-dependent HR-like symptoms in XopJ expressing leaves requires interaction of the effector protein with its virulence target RPT6. In order to investigate whether SA treatment would affect the ability of XopJ to degrade RPT6, protein degradation was assessed in leaves co-expressing both proteins after treatment with SA versus the control (Supplementary Figure S4). However, no difference in RPT6 protein amount could be detected between SA treated leaves and the control. Thus, SA treatment *per se* does not influence RPT6 degradation by XopJ.

### The Role of NPR1

**FIGURE 4:**
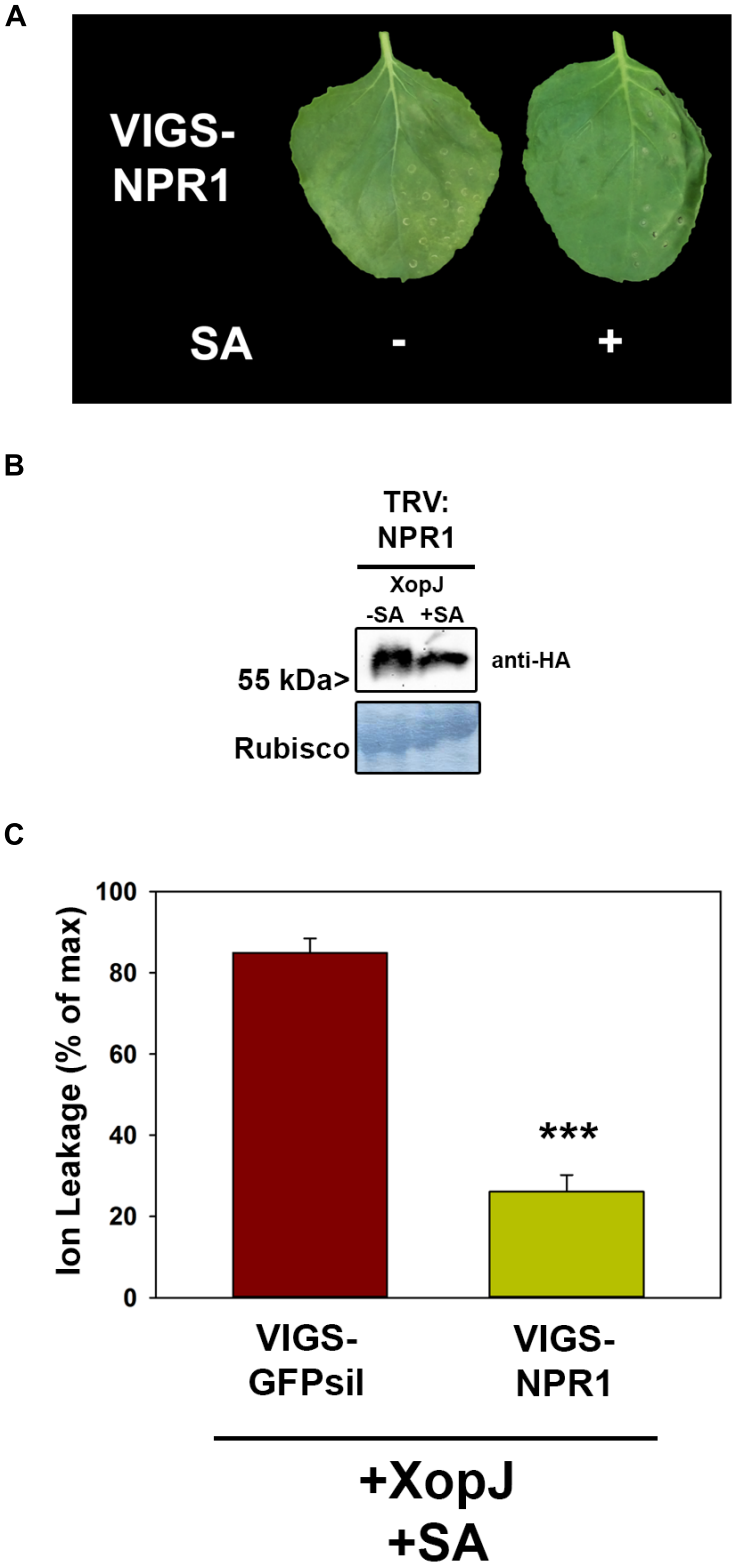
**VIGS of NPR1 prevents XopJ induced HR-like cell death after SA treatment. (A)** XopJ was transiently expressed in NPR1 silenced plants and treated with 5 mM SA 24 hpi. Leaf phenotypes were photographed 48 hpi. **(B)** Protein extracts from TRV:NPR1 leaves transiently expressing XopJ-HA treated with or without 5 mM SA. Amido black staining served as a loading control. **(C)** Ion leakage was determined in TRV:NPR1 and TRV:GFPsil plants transiently expressing XopJ following SA treatment. Bars represent the average ion leakage measured for triplicates of six leaf155 disks each, and the error bars indicate SD. Significant differences are indicated by asterisks (***P < 0.001). The experiment was repeated three times with similar results.

The experiments described above suggest a role of SA and SA-signaling in the phenomenon of SA-dependent HR-like symptom elicitation by XopJ. Thus, we sought to investigate the contribution of a SA signaling component to the process. NPR1 (non-expressor of PR1) is a key positive regulator of SA-mediated defense responses notably by activating transcription of a battery of genes in response to rising SA-levels (Pieterse and Van Loon, 2004). Down-regulation by VIGS in *N. benthamiana* plants was used investigate the involvement of SA-signaling via NPR1 with GFPsil serving as a control. Two weeks after TRV inoculation leaves of silenced plants were infiltrated with *Agrobacteria* harboring XopJ-HA and 24 hpi infiltrated leaves were sprayed with SA. As shown in **Figure 4A**, NPR1 silenced plants did not develop visible signs of HR-like symptoms upon SA treatment of XopJ infiltrated leaves, suggesting a critical role of NPR1 in execution of this response. Western blot analysis showed that XopJ was expressed in VIGS-NPR1 leaves with or without SA-treatment (**Figure 4B**). Measurement of ion leakage confirmed the observed phenotype, as conductivity in NPR1 silenced plants was significantly lower compared to TRV:GFPsil control plants following SA-treatment (**Figure 4C**). Thus, NPR1 appears to be essential for XopJ to trigger HR-like symptoms upon SA-treatment.

### XopJ’s Inhibitory Effect on the Proteasome is Affected by SA-Treatment and Requires NPR1

**FIGURE 5:**
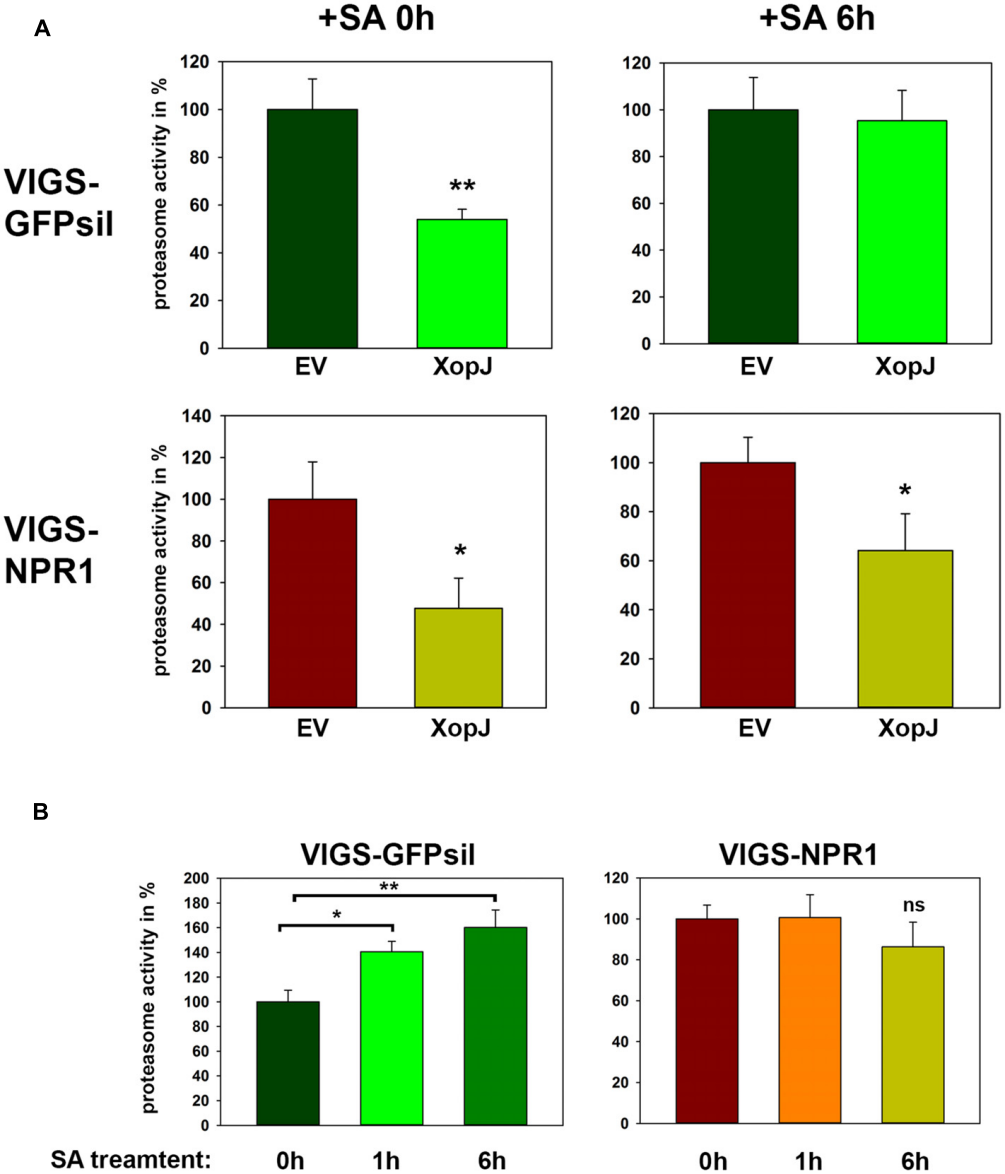
**SA treatment interferes with XopJ’s ability to suppress proteasome activity in a NPR1-dependent manner. (A)** XopJ along with an EV control were transiently expressed in leaves of TRV:GFPsil and TRV:NPR1 leaves using Agro-infiltration. After 48 h, leaves were sprayed with 5 mM SA and relative proteasome activity in total protein extracts was determined at 0 and 6 h post SA treatment by monitoring the breakdown of the fluorogenic peptide Suc-LLVY-AMC. The EV control was set to 100%. Data represent the mean SD (*n* = 3). The asterisk indicates a significant difference (*P < 0.05; **P < 0.01) based on results of a Student’s *t*-test. **(B)** VIGS NPR1 and GFPsil control leaves were sprayed with 5 mM SA and proteasome activity in total leaf extracts was determined at time points indicated in the figure by monitoring the breakdown of the fluorogenic peptide Suc-LLVY-AMC. Data represent the mean SD (*n* = 3) Significant differences are indicated by asterisks (*P < 0.05; **P < 0.01) and were calculated using Student’s *t*-test. (ns = not significant). The experiment has repeated three times with similar results.

XopJ has been shown to dampen proteasome activity during the compatible interaction of Xcv with pepper plants and this leads to a delay in the development of host cell necrosis (Üstün et al., 2013). Further analysis revealed that this effect was dependent on NPR1, as proteasome activity seems to be partially regulated by NPR1 during defense (Üstün et al., 2013). In the light of these observations, we next investigated whether the inhibitory effect on the proteasome is maintained when XopJ expressing plants are treated with SA and whether the associated changes are NPR1 dependent. To circumvent the negative effect of SA-mediated HR-like cell death on the overall proteasome function, we monitored proteasome activity 6 h after spraying *N. benthamiana* leaves expressing either EV or XopJ with SA, as previous results showed that the proteasome is activated upon SA treatment reaching a peak at 6 h after SA-treatment (Üstün et al., 2013). After transient expression of XopJ or EV and subsequent SA-treatment for 6 h, proteasome activity was measured in NPR1 silenced and GFPsil control plants. Spraying plants with SA led to a loss of XopJ’s ability to inhibit the proteasome in control plants, whereas in NPR1 silenced plants XopJ was still able to suppress proteasome activity 6 h after SA-treatment (**Figure 5A**). To show that the SA-dependent activation of the proteasome function might be affected in NPR1 silenced plants, proteasome activity was determined in TRV:GFPsil and TRV:NPR1 treated with SA for 1 and 6 h, respectively. In accordance with previous findings, SA significantly elevated proteasome activity in GFPsil control plants but not in *NPR1* silenced plants (**Figure 5B**). These data suggest that the ability of XopJ to interfere with the proteasome function can be counteracted by the exogenous application of SA.

## Discussion

Depending on the genetic context of the plant with which a given bacterial pathovar interacts, T3Es can either act as virulence factors or, upon recognition by cognate R proteins, may function as Avr factors which then trigger a strong defense response typically characterized by an HR (Dodds and Rathjen, 2010). In this study, we investigated the Avr function of T3E XopJ from Xcv in the non-host plant *N. benthamiana* and demonstrated that the effector is able to trigger HR-like symptoms when transiently XopJ expressing tissue is treated with SA. We have previously characterized the virulence activity of XopJ and could show that it acts as a protease to degrade the proteasomal subunit RPT6 which subsequently leads to the inhibition of proteasome activity in host cells. Reduced proteasomal protein turnover interferes with SA-mediated defense responses as well as vesicle trafficking and attenuates host-induced necrosis during infection of susceptible pepper plants (Bartetzko et al., 2009; Üstün et al., 2013; Üstün and Börnke, 2015). Many of the defense responses that XopJ interferes with depend on the central SA-signaling component NPR1 and XopJ-mediated inhibition of the proteasome appears to interfere with proper NPR1 function (Üstün et al., 2013; Üstün and Börnke, 2015).

Like other members of the YopJ-family of effector proteins XopJ possess a catalytic triad that is required for its protease activity and is also essential for its virulence function (Üstün et al., 2013; Üstün and Börnke, 2015). Mutant studies revealed that the Avr activities of the YopJ-family members from plant pathogens depend on the catalytic triad, suggesting that the enzymatic function is required for the recognition by corresponding plant R proteins (Orth et al., 2000; Roden et al., 2004; Bonshtien et al., 2005; Whalen et al., 2008). The fact that the XopJ-induced, SA-dependent HR-like symptom development also requires the catalytic cysteine residue C235 indicates that XopJ is recognized indirectly via its enzymatic activity. This is similar to the *Pseudomonas syringae* T3Es AvrRpt2 and HopAR1 (formerly AvrPphB) which are recognized by their cognate R proteins in *Arabidopsis* via their protease activity (Axtell et al., 2003; Shao et al., 2003; Ade et al., 2007). The resistance protein RPS5 recognizes the proteolytic degradation of the HopAR1 target protein PBS1, while RPS2 is activated upon cleavage of the host target protein RIN4 by AvrRpt2. Silencing of the XopJ host target protein RPT6 prevents development of HR-like symptoms after SA-treatment further supporting the notion that XopJ is recognized indirectly via its proteolytic activity on its host target protein. Furthermore, the predicted myristoylation site of XopJ that localizes the protein to the host cell plasma membrane is also required to induce an SA-dependent HR. Mutation of the myristoylation site of XopJ has previously been shown to abolish its virulence function, indicating that plasma membrane localization inside the host cell is required for both activities. Thus, the data support a model in which XopJ’s ability to elicit HR-like symptoms in *N. benthamiana* is closely linked to its virulence target in host plants and in that RPT6 is guarded by a yet unknown R protein whose activation requires two signals (1) degradation of RPT6 and (2) activation of SA-signaling. Previous evidence suggests that RPT2a and RPT2b, two isoforms of another subunit of the proteasomal RP19, interact with the CC-NBS-LRR protein uni-1D from *Arabidopsis* and that this interaction is involved in triggering uni-1D-induced defense signaling (Chung and Tasaka, 2011). Hence, the uni-1D/RPT2 interaction provides an example of a proteasomal subunit that appears to be guarded by an R protein. Therefore, it could be well possible that the same is true for RPT6 in *N. benthamiana*.

XopJ-induced development of HR-like symptoms upon SA-treatment was dependent on SGT1, which is required for resistance mediated by multiple R proteins recognizing a diverse set of pathogens. SGT1 has been shown to control the steady-state level of preactivated R proteins (Peart et al., 2002; Azevedo et al., 2006). Virus-induced silencing of SGT1 in *N. benthamiana* considerably reduced HR-like symptom development in XopJ expressing leaves upon SA-treatment, suggesting the involvement of R-protein-mediated signaling in this process. The resistance protein responsible for the recognition of XopJ in *N. benthamiana* remains to be identified. Based on the finding that silencing of NDR1 strongly reduces development of XopJ-mediated HR-like symptoms, it might be assumed that the R protein associated with these responses belongs to the CC-NBS-LRR class (Aarts et al., 1998), similar to what has been described for the RPT2/uni-1D couple in *Arabidopsis* (Chung and Tasaka, 2011).

Although a large body of evidence suggests a central role of SA and SA-signaling in the elicitation of cell death during HR (Vlot et al., 2009), it remains unclear why XopJ-triggered HR-like symptoms in *N. benthamiana* depend on the exogenous application of SA to XopJ expressing leaves. Exogenously applied SA appears to trigger the canonical SA-signaling pathway that operates via NPR1 as the central regulator. Plants silenced for NPR1 expression lose the ability to elicit HR-like symptoms upon SA treatment of XopJ expressing leaves. We could show that SA treatment induces proteasome activity in an NPR1 dependent manner and that in the presences of SA XopJ is no longer able to inhibit the proteasome. The reason for this phenomenon is currently unclear but a possible explanation could be that either the induction of proteasome activity by SA is quantitatively stronger than XopJ’s ability for its inhibition or that SA can directly interfere with the ability of the effector to degrade RPT6. However, western blot experiments suggest that SA treatment has no effect on the ability of XopJ to degrade RPT6 in transient expression assays. Thus, activation of the proteasome beyond a certain threshold in the presence of a functional XopJ protein could act as a signal for R protein activation. HR-like symptom elicitation by XopJ might follow a two-signal model in which the first signal is the degradation of the host cell protein RPT6 by XopJ and the second signal is provided by elevated SA levels. A similar model has previously been proposed for the *Pseudomonas syringae* T3Es AvrE and HopM1 (Lindeberg et al., 2012). According to this model AvrE or HopM1 trigger ETI by interfering with a process, e.g., vesicle trafficking, rather than with a specific protein. Plants then reduce spurious cell death responses resulting from vesicle trafficking perturbations by requiring a second signal such as increases in SA to eventually trigger ETI (Lindeberg et al., 2012). For XopJ this would mean that the sole inhibition of proteasomal turnover by removal of RPT6 would not be interpreted as a danger signal by the plant immune system but cell death is only triggered when there is a concomitant rise in SA contents. Alternatively, expression of the R protein required to recognize XopJ action on RPT6 could be dependent on SA as has previously been shown for the R proteins RPW8.1 and RPW8.2 in *Arabidopsis* which show induction on the transcriptional level after exogenous application of SA (Xiao et al., 2003).

Since we used transient expression of XopJ by *Agrobacterium*-infiltration we currently cannot make any statement about the role of XopJ in triggering HR-like symptoms during an incompatible interaction of Xcv with *N. benthamiana*. When inoculated with a high titer into *N. benthamiana* leaves Xcv has been shown to elicit plant cell death (Metz et al., 2005). The T3E responsible for this host response has been identified as XopX. Interestingly, the visual cell death response phenotype was not elicited by *Agrobacterium*-mediated expression of XopX. However, a cell death response could be elicited if the *Agrobacterium*-mediated XopX expression was co-inoculated with XopX deficient Xcv or with *Xanthomonas campestris* pv. *campestris* that carry a functional T3SS (Metz et al., 2005). XopX has recently been proposed to interfere with PTI responses to promote Xcv virulence (Stork et al., 2015). Thus, XopX triggered cell death responses would also follow a two-signal model in which the second signal is dependent on a functional T3SS (Metz et al., 2005; Stork et al., 2015).

## Conclusion

**FIGURE 6:**
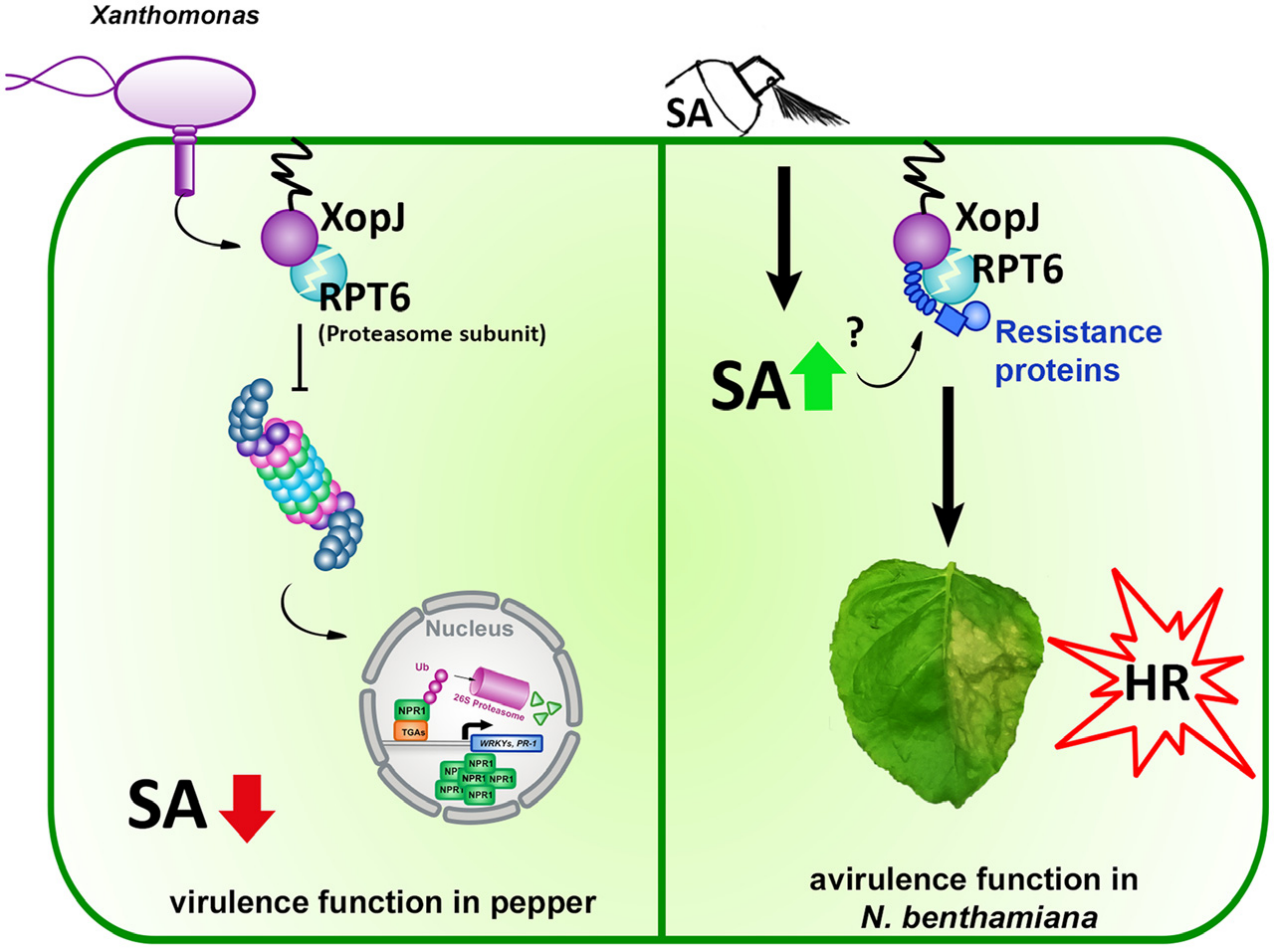
**Model of XopJ’s virulence and avirulence (Avr) function in a host and non-host plant, respectively. (Left)** pathogenic Xcv injects XopJ via its type-III secretion system into pepper host cells where it cleaves RPT6 to inhibit the proteasome. Reduced proteasomal activity interferes with the turnover of defense components, suppresses SA accumulation and results in the attenuation of SA-mediated defense responses. **(Right)** In the non-host plant *N. benthamiana* transiently expressed XopJ cleaves endogenous RPT6. When SA levels are increased by exogenous application of the defense hormone, R-protein mediated recognition of RPT6 cleavage by XopJ is activated leading to the induction of HR-like symptoms.

We could show that XopJ’s ability to trigger an SA-dependent HR-like host response is tightly linked to its virulence function (**Figure 6**) and provide another example, in addition to the previously described T3Es XopX, AvrE, and HopM1, for an effector following a two-signal model to elicit a defense response in plants.

## Conflict of Interest Statement

The authors declare that the research was conducted in the absence of any commercial or financial relationships that could be construed as a potential conflict of interest.
