# Supplementary material for: The Xanthomonas effector XopJ triggers a conditional hypersensitive response upon treatment of N. benthamiana leaves with salicylic acid
Source: Front Plant Sci. 2015 Aug 3;6:599. doi: 10.3389/fpls.2015.00599 (PMC4522559; doi:10.3389/fpls.2015.00599)
Supplement: Supplementary file 1 [file Data_Sheet_1.PDF]

Supplementary Figures to Üstün et al. "The *Xanthomonas* effector XopJ triggers a conditional hypersensitive response upon treatment of *N. benthamiana* leaves with salicylic acid"

Supplementary Figure S1.

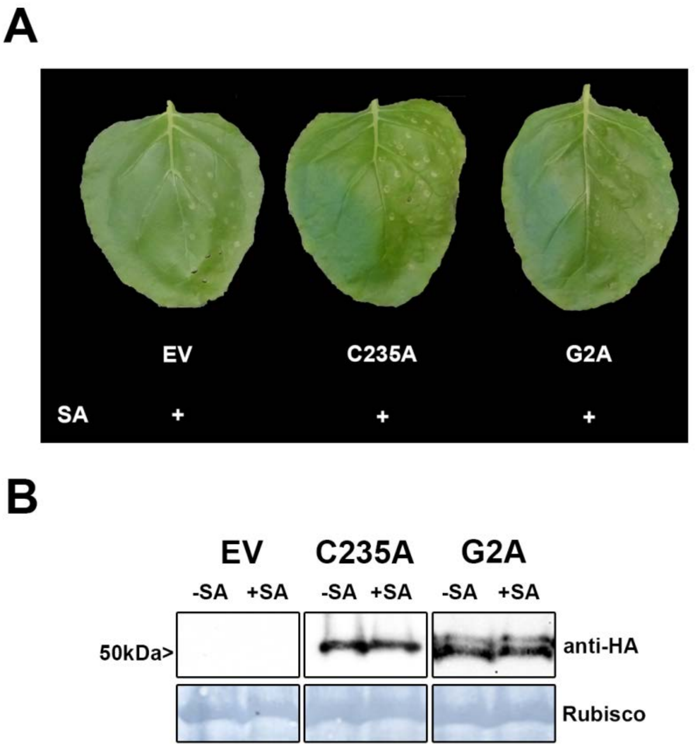

Figure S1: XopJ-induced HR-like phenotype after SA treatment is dependent on its myristoylation and catalytic activity. (A) Phenotype of *N. benthamiana* leaves (one half) infiltrated with *Agrobacterium tumefaciens* strains that mediate T-DNA-based transfer of XopJ G2A-HA, XopJ C235A-HA and empty vector (EV). Leaves were sprayed with 5 mM salicylic acid (SA) 24 h post infiltration (hpi) and leaves were photographed 48 hpi. (B) Protein extracts from *N. benthamiana* leaves transiently expressing XopJ G2A-HA, XopJ C235A-HA and empty vector (EV) at 48 hpi were prepared. Equal volumes representing approximately equal protein amounts of each extract were immunoblotted and proteins were detected using anti-HA antiserum. Amido black staining served as a loading control.

Supplementary Figure S2.

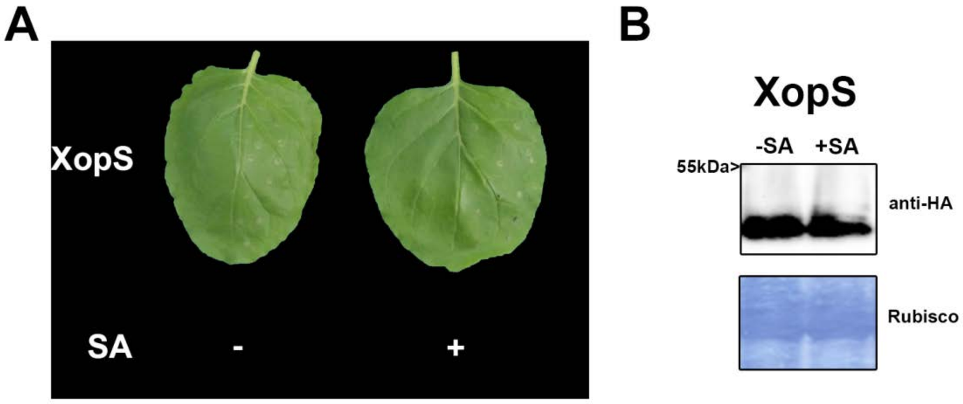

Figure S2: XopS does not induce cell death after SA application. (A) XopS-HA was transiently expressed in *N. benthamiana* leaves and treated with 5mM SA or water. Picture was taken 48 hpi. (B) Western blot analysis confirming proper XopS expression in all conditions using an anti-HA antibody.

Supplementary Figure S3.

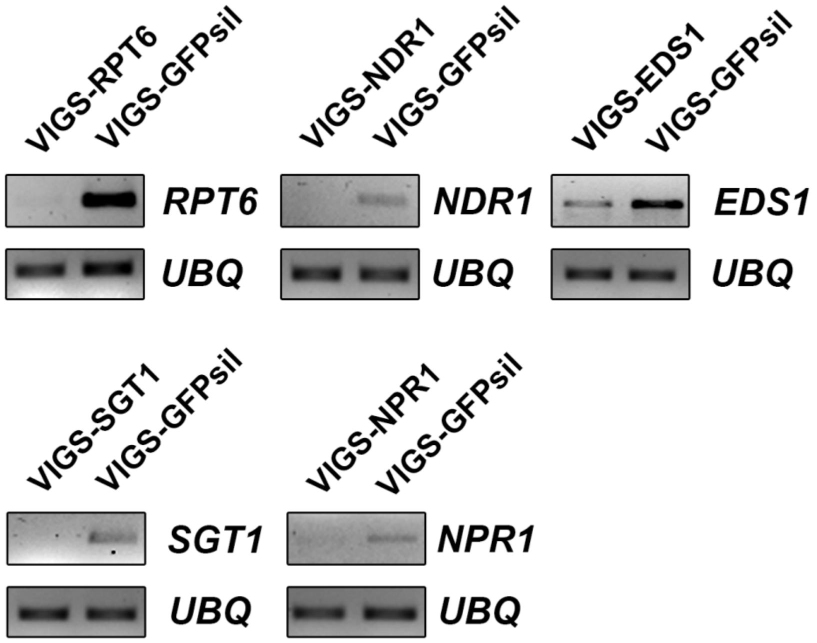

Figure S3: Virus-induced gene silencing of immunity components. Transcript accumulation of *RPT6, NDR1, EDS1, SGT1* and *NPR1* and ubiquitin (*UBQ*) in VIGS-RPT6, VIGS-NDR1, VIGS-EDS1, VIGS-SGT1, VIGS-NPR1 and control plants (VIGS-GFPsil) was analyzed by RT-PCR. *UBQ* served as an internal control.

Supplementary Figure S4.

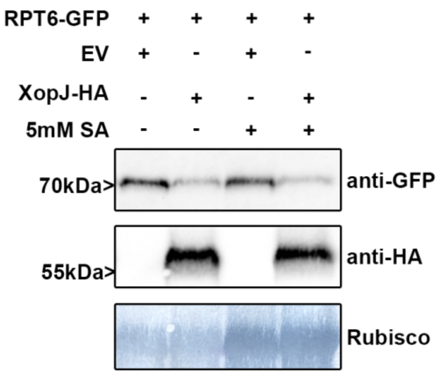

Figure S4: SA application does not affect XopJ-mediated RPT6 degradation. RPT6-GFP together with EV or XopJ-HA was transiently co-expressed in *N. benthamiana* using *A. tumefaciens*. Application of 5mM SA (absence or presence indicated in figure with + or -) was performed 42 hpi and total proteins were extracted 6 hours later. RPT6-GFP protein levels were detected using an anti-GFP antibody. Expression of XopJ was verified using an anti-HA antibody after stripping the same membrane. Staining of the membrane with amido black showed equal loading.
